# Extraction and quantification of lead in meconium: Analysis through inductively coupled plasma–mass spectrometer (ICP-MS)

**DOI:** 10.1016/j.mex.2026.103857

**Published:** 2026-03-11

**Authors:** Sharmind Neelotpol, Alastair Hay, Mike Woolridge

**Affiliations:** a Professor, School of Pharmacy, BRAC University, Bangladesh; b Professor (Emeritus) of Environmental Toxicology, Division of Epidemiology, University of Leeds, UK; c Independent Researcher, Harrogate, North Yorkshire, UK

**Keywords:** Extraction, Lead, ICP-MS, Meconium, Calculation, Recovery rate

## Abstract

In recent years, growing attention has been directed towards the impact of environmental pollution on child health, particularly the silent threat posed by heavy metals (HMs). Foetuses may also be exposed to HMs through placental transfer from the mother. To evaluate such exposures, researchers are increasingly seeking long-term, non-invasive biomatrices, among which meconium - the newborn’s first faeces or stool, has emerged as a promising candidate. Meconium accumulates throughout pregnancy and is likely to reflect the transfer of chemicals across the placenta. A total of 136 meconium samples were analysed using ICP-MS to evaluate lead concentration in it. The method achieved a detection limit of 0.00062 µg/g (0.62 ppb). The mean recovery rate of Pb was 101% (range: 93–110%). This study presents the following challenges and solutions:

• Extraction, digestion, and quantification of lead (Pb) in human meconium.

• Identification of appropriate drying time of meconium samples due to variable water content across samples.

• Spiking of meconium with suitable Pb standards, calculation of recovery rates, and estimation of true Pb concentration.

## Graphical abstract

**Figure:**
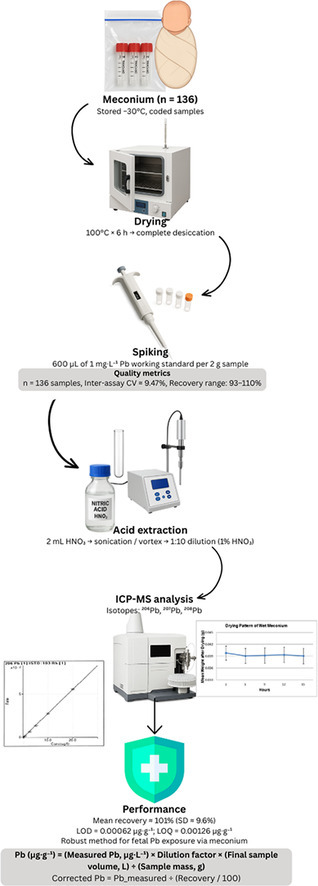

**Specifications table**

**Table:** 

**Subject area**	Agricultural and Biological Sciences
**More specific subject area**	Measurement of xenobiotics, including neurotoxicants
**Name of your method**	This study described the extraction process of lead (Pb) in meconium and its analysis by ICP-MS Method
**Name and reference of original method**	None
**Resource availability**	Not applicable

## Background

Lead is prevalent in the environment and its exposure occurs from many sources e.g. lead based paint in older homes, contaminated soil and dust, water pipes (lead leaching from older corroding pipes), inhalation in industrial settings, dermal contact, herbal and traditional remedies, ethnic make-up and lead glazed pottery [1]. Children, in particular, may also be exposed to lead ingestion from the habit of pica of lead-based paint, which is peeling or flaking.

A mother’s exposure to lead through her lifestyle will result in lead being passed across the placenta to her foetus during gestation [2,3]. Lead can freely cross placenta by diffusion and may be a cause of premature rupture of membrane [4]; preterm delivery [5]; lower mean gestational age [6]; low birth weight [7]; effect on the foetal brain [8] or even foetal death in extreme cases [9,10].

Various bio-matrices, including blood, umbilical cord blood, and placenta have been employed to measure environmental toxins in the foetus [11]. Researchers are increasingly interested in exploring biological markers which can be collected by non-invasive means. In this context, meconium - baby's first faeces or stool, composed of water (∼ 80%), mucopolysaccharides, bilirubin, intestinal enzymes, hair and squamous cells, which is viscous, odorless and tar-like discharge within two to three days after birth, is a very useful biological matrix. This can be used to examine cumulative prenatal exposure, because what the foetus swallows throughout gestation can either be incorporated into the foetal skeleton or excreted in meconium [12]. This excretory waste represents an integrated estimate of cumulative foetal exposure which can be quantified non-intrusively following its excretion after birth. Meconium, therefore, provides a wider opportunity to detect substance exposure in the foetus and serve as a repository for a variety of metabolites including xenobiotics, and environmental contaminants [[13], [14], [15]].

Limited studies focused on meconium as biomatrix of lead. We have identified studies which analysed lead (Pb) in meconium [16] using ICP-MS method; however, their study did not describe details of the extraction process of lead from meconium. There are various methods for the analysis of lead in meconium; however, ICP-MS is the most exquisitely sensitive and reliable method for the measurement of metals. Previously, lead in meconium has been analyzed by Graphite Furnace Atomic Absorption Spectrometry (GFAAS) [17], Furan Atomic Absorption Spectrometry (FAAS) [15], Atomic Absorption Spectrometry (AAS) [18]. Inductively Coupled Plasma Mass Spectrometry (ICP-MS) is a more precise and accurate instrument than the previously used MS or GFAAS methods [19]. Recently Gundacker *et al* (2010) [20] analyzed lead in meconium by High Performance Liquid Chromatography – Cold Vapour – Inductively-Coupled Plasma Mass Spectrometry (HPLC-CV-ICPMS). No studies have been identified, however, which have developed, validated or adopted ICP-MS to analyze lead in meconium.

This is the first detailed study describing the extraction and measurement process of lead from meconium using ICP-MS method. This study explains the steps involved in the lead extraction process including the appropriate drying time of meconium, as water content varies among the samples; the process of spiking meconium with an appropriate lead standard; and calculation of recovery rate and true concentration of lead in meconium. Therefore, it can be said that, because of high sensitivity, this study may be considered by the researchers who are interested to work in this field.

## Method details

### Materials and Reagents

For this study, recruitment of the participants and collection method of meconium samples were described by Neelotpol et al., (2016) [21]. Moreover, in western cultures there are further practical issues surrounding the collection of meconium from babies after their mothers have been discharged early from the maternity unit; these problems have also previously been addressed in the same article [21]. Individual or pooled samples (serial meconium samples from the same baby) can be used for the evaluation of exposure to contaminants, as well to the possible historical depositions of these compounds during pregnancy. However, pooled samples were used for this study because, irregular exposure was studied, resulting in irregular chemical deposition [22]. Collected meconium samples were stored at -30°C in a vinyl bag with an identification code [17]. Glass materials, nitric acid, lead isotope solution, lead standard, and de-ionized water were required for this analysis. Nitric acid (Aristar (BDH); Pb <0.002 ppm), lead isotope solution (^206^Pb ^207^Pb ^208^Pb), and lead standard (1000 mg/l) obtained from VWR International (LLC, catalogue number 14036) were purchased. De-ionized water was prepared in the laboratory under Faculty of Medicine, University of Leeds, UK.

#### Equipment cleaning and preparation

Prior to analysis it was important to make sure that all glassware and flasks were free from lead. Flasks (50ml), glass tubes (75 mm), and polypropylene tubes (12×50 mm) were washed and soaked overnight in 3% nitric acid, with all efforts made to ensure that all components were fully submerged in the acid. Tubes and flasks were rinsed six times with de-ionised water and the items allowed to air dry within a closed drying chamber.

### Optimization and findings during method development to measure lead in meconium

Optimization of the method for the analysis of lead in meconium: Compared to liquids, preparation of solid samples for the measurement through ICP-MS is more complex as they are required to be in solution before analysis. In order to develop a method for the analysis of lead in meconium, various steps were explored and established, including removal of a given weight of sample, drying of meconium and the optimal volume of nitric acid required to ensure accurate extraction of lead from meconium, to prepare an appropriate substrate for assay by ICP-MS.

During optimization, one hundred milligrams of meconium was dissolved in different volumes of concentrated nitric acid (HNO_3_) (manufacturer declared purity >69%) and mixed vigorously by vortexing in order to determine what amount of nitric acid resulted in the fullest extraction of lead from meconium. Another trial was performed in which volumes of between 5 to 10ml of nitric acid were used, with 20-30 min being allowed for digestion of the meconium matrix. Samples were then evaporated by ignition to reduce the volume to 1-2 ml, then filtered and assayed. Before analysis each samples were again mixed by vortexing.

It was also important to compare whether there were differences in lead content in identical meconium samples when held in either glass or plastic tubes. Accordingly, meconium was dissolved in HNO_3_, in both glass and plastic tubes to test this possibility. Any differences detected would suggest either leaching from, or absorption of lead by the container.

**Fig. 1:**
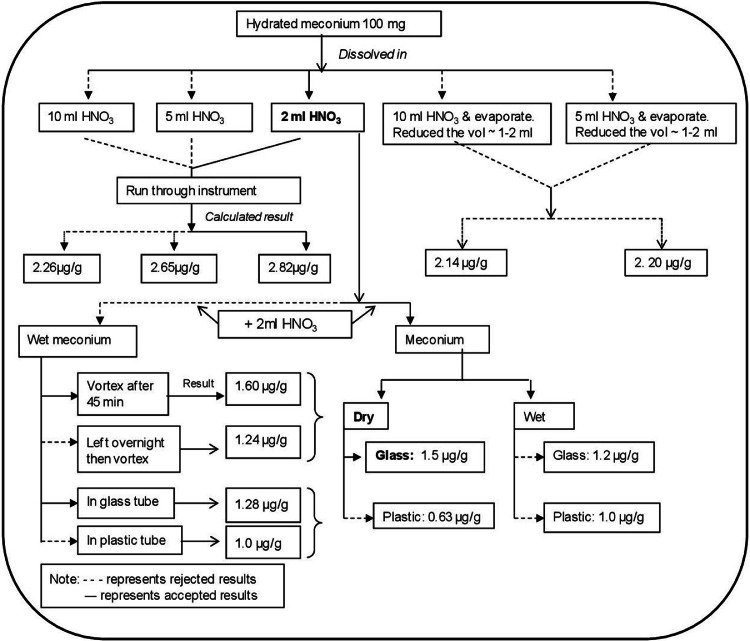
Flow chart summarizing steps taken to optimize the assay for lead in meconium by ICP-MS.

A range of the lead concentrations was used (0.25, 0.5, 1.5, 2.5, 5, 10, and 20 µg/l Pb), including low and high points on the calibration curve for lead isotopes, anticipated to encompass the range of lead values to be found in meconium. A defined amount of meconium (2g) was spiked with 400 µl, 600 µl and 800 µl of lead standard (1 mg/l) in order to calculate the recovery rate of lead in meconium. All the measures taken to optimize the method for the analysis of lead in meconium are illustrated in Fig. 1.

### Calibration using lead standards

**Fig. 2:**
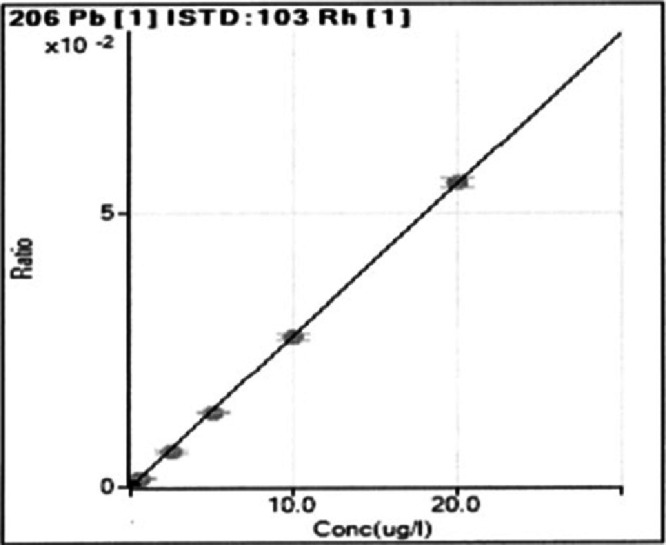
Representative standard curve for the concentration of lead.

A certified lead standard solution was used to calibrate the assay. A ‘Stock Solution’ of 1000 mg/l of lead in 2% HNO_3_ was made and a working solution (WS) of 1mg/l was prepared. The lead content of meconium was determined from a calibration curve, generated using an aqueous lead standard dissolved in the same concentration of acid as the samples and internal standards on each occasion the assay was performed. Lead standards, from a working solution of 1mg/l were prepared in 65% nitric acid at concentrations of 0.25-20 μg/L. A representative calibration curve used in this study is shown in Fig. 2.

### Preparation of meconium samples

*Spiking meconium with lead:* It is essential to establish how efficient the recovery is of the substance of interest from the sample, using known concentrations of that substance. This is known as the ‘recovery rate’ and represents the accuracy of the measurements of lead made from meconium. Meconium is a viscous mucoid solid that needs to be solubilised. Sample viscosity was reduced by the addition of lead standard solution in HNO_3_. The volume of lead standard solution added proved to be a critical step, ensuring there was enough to allow efficient mixing of the meconium and distribution of the lead standard solution throughout the sample. Initially, separate 2g samples of meconium were spiked with either 400µl, 600µl or 800µl of lead working solution (1mg/l) to ensure the meconium sample changed from viscous to free-flowing.

Meconium spiked with 400 µl of working solution remained too viscous to mix effectively, while that spiked with 600µl of lead working solution gave a free flowing mixture throughout the sample. An 800 µl addition of lead WS to the 2 g of meconium sample gave semi liquid property to the sample. As a consequence, a compromise of 600µl working solution of lead was used to spike 2g of meconium.

*Drying of meconium:* Initial observations with six meconium samples revealed substantial variation in the moisture content between samples. If analytical values were to be reported per unit weight of sample, then variation in moisture would lead to analytical error. Therefore, samples were dried prior to analysis and the optimal time and temperature required to achieve desiccation was evaluated. Lead content could then be expressed in units of grams per gram (‘dry weight’ of meconium) in order to provide a standardized measure, more readily comparable with other studies. Six different samples were processed in triplicate on three separate occasions using different duration of time and temperature.

To an acid-washed glass specimen tube, a hydrated sample of 200mg of meconium was placed into a heat block at different temperature and time (at either 50°C, 80°C, 100°C for 3, 6, 9, 12, 15, and 24 hours) until dry and a consistent weight was achieved. The evaporated water content of the sample was then calculated. Maximum moisture loss was achieved with drying at 100°C for 6 hours, with negligible further losses from longer periods of drying at this temperature. However, even with these drying parameters, the moisture content of samples varied substantially, so that moisture loss could range from 29% to 80%. Nonetheless, 6 hours drying at 100°C was sufficient to ensure complete desiccation, so this procedure was adopted as being both consistent and reliable, achieving drying on a practicable timescale for performing the assay.

**Fig. 3:**
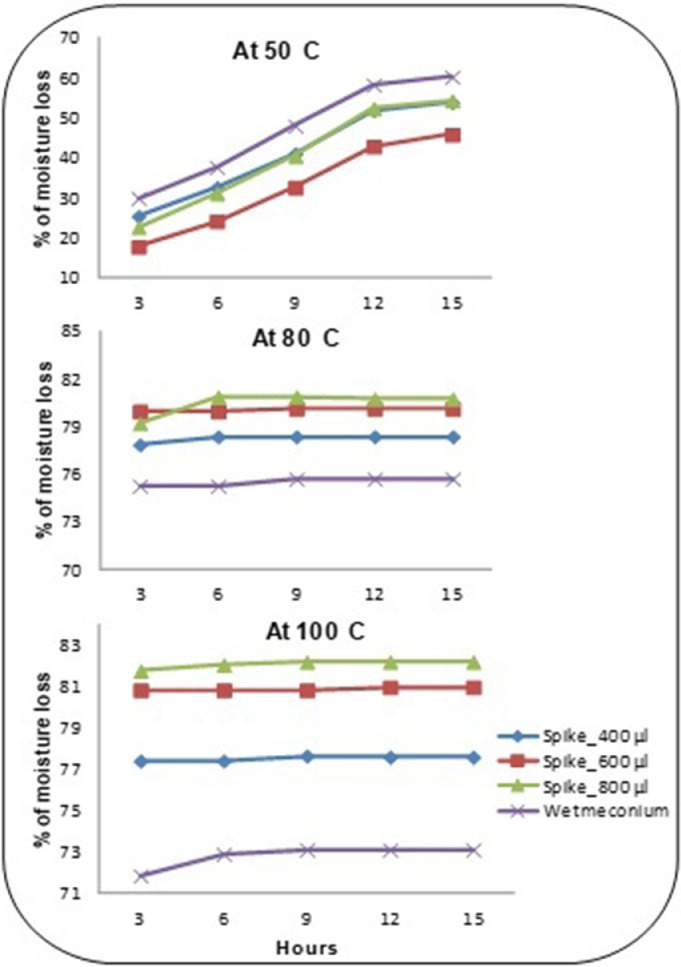
Effect of time and temperature upon water loss from meconium samples.

**Fig. 4:**
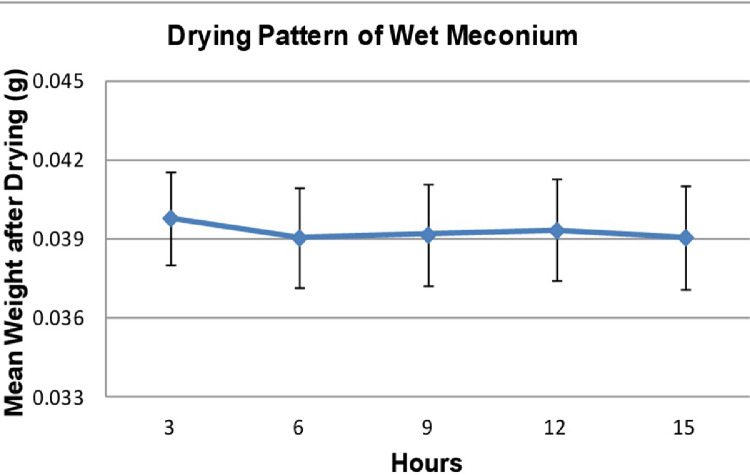
Weight change of meconium samples dried at 100°C for 3-15 hours; error bars indicate ± SD (n=6).

When lead is measured by ICP-MS, it is not necessary to desiccate the substrate to dry ash (dry ashing eliminates or minimizes the effect of organic materials in mineral element determination). This laboratory method only required the sample to be desiccated to evaporate the maximum amount of moisture, without removing organic fats and oils, which do not interfere with the assay. The mean loss of moisture from six meconium samples at different temperatures and time intervals is illustrated by Fig. 3, Fig. 4 respectively.

### Analysis of meconium by ICP-MS

**Fig. 5:**
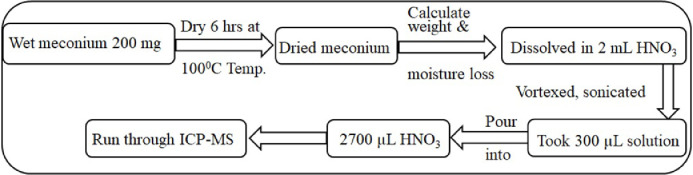
Flow diagram of the extraction of lead in meconium by ICP-MS.

On the day of analysis, samples were thawed to room temperature and thoroughly mixed in order to minimise differences in lead content within the mass of the original meconium sample. A sample of 200mg was placed into a glass container, placed in a heat block and left at 100°C for 6 hours. The dried sample was suspended in 2 ml HNO_3_, vortexed and sonicated briefly, to suspend all the sample. Nitric acid solution (1%) was added to the extracted solution in a 1:10 ratio (sample: HNO_3_ (1%)). This final solution was aspirated (2 µl) into an Agilent 7700 ICPMS instrument for analysis. The procedure for measuring the concentration of lead in meconium is illustrated by Fig. 5.

## Method validation

### Calculation with example

1. Calculation of true lead in meconium sample:

1.1 Calculation of recovery rate:

Identifying the recovery rate is crucial in order to gain a true measure of the lead concentration by unit dry weight of sample. The method of extraction can be regarded as satisfactory, when a high recovery rate is achieved. Following the process of analysis outlined above, the mean recovery rate calculated was 101% (min-max: 93-110%). To calculate the true lead level in meconium certain calculations need to be undertaken. Only after correcting the calculated lead value for the recovery rate, it is possible to calculate the true concentration of lead in meconium.

In this method, the limit of quantification (LOQ) and the detection limit were determined as ten times and three times the values of the standard deviation calculated from the analysis of ten blank samples, respectively. To measure the true lead level in meconium, following calculations were undertaken:

(1) Calculation of lead (Pb) in meconium: Equation No.

(a) Mean of analysed Pb value (isotope ^206^Pb, ^207^Pb, ^208^Pb) x 10 µg/l … … I
(b) I x 2/1000 µg/l … … … … … … … … … … … … … … … … … … … II
(c) II x 1000/weight of dried meconium µg/g … … … … … … … … … … III

For example:

(a) Mean of analysed Pb value (isotopes ^206^Pb, ^207^Pb, ^208^Pb): 3.84 µg/l
(b) Volume of HNO_3_: 2ml
(c) Weight of dried meconium: 59.7 mg. Results were expressed in µg/g.

The amount of lead in the meconium sample was then calculated using the above formulae:

Due to the 10 times dilution of the preparation ―

(3.84×10) µg/l = 38.4 µg/l … … … … … … … … … … …. (I)

As it was dissolved in 2 ml HNO_3_ ―

38.4×2/1000 = 0.0768 µg/l … … … … … … … … … … … (II)

Therefore: 59.7 mg meconium contains ―

0.0768×1000/59.7 = 1.2864 µg/g of lead … … … … … … .. (III)

(2) Calculation of Recovery Rate:

A. Calculation of the expected concentration of Pb in the spiked meconium

Taken Pb Std (µg) X weight of wet meconium (mg) taken … … IV

Wet weight of spiked meconium (µg)

IV x 1000/2 µg/l … … … … … … … … … … … … … … … … V

B. Recovery rate:

(Analyzed spiked sample - Analyzed un-spiked sample)/ V … … VI

VI x 100 … … … … … … … … … … … … … … … … … … VII

For example:

A. Calculation of the expected lead from the spiked meconium

(a) Lead Std (µg): 600 µl

(b) Weight of wet meconium: 165.9 mg

(c) Wet weight of spiked meconium: 2.024 g

The calculation is ―

0.6×165.9/2624 = 0.0379 µg/2ml (as it was dissolved in 2ml HNO_3_)

= 0.0189 µg/ml … … … … … … … … … … … (IV)

= 18.96 µg/l… … … … … … … … … … … … (V)

B. Recovery rate:

(a) Analyzed result of spiked meconium: 24.7 µg/l
(b) Analysed result of unspiked meconium: 3.86 µg/l

Therefore, the recovery rate is {(a - b) / V} X 100 i.e. {20.9 / 18.96} X 100 = 1.10 µg/l X 100 = 110% … … … … … … (VI & VII)

(3) Calculation of True Pb:

(III / VII) x 100 … … … … … … … … … … … … … … … … … VIII

For example:

To calculate true lead:

(a) Result of analyzed lead i.e. (III): 1.28 µg/g
(b) Result of recovery rate i.e. (VII): 110%. Results were expressed in µg/g.

(1.28 / 110) X 100 = 1.16 µg/g

• Following the above calculation, the true lead in the meconium sample was 1.16 µg/g. Using the above method with 136 meconium samples, the mean recovery rates assayed in triplicate on three separate days was 100.854% (range: 93.11-110.12), SD: 9.549; Interassay CV%: 9.468. The calculated limit of detection was 0.00062µg/g and the limit of quantification was 0.00126µg/g. Scientists found ICP-MS a more precise and accurate instrument than the previously used methods [23]. Therefore, this study proves ICP-MS as a sophisticated, highly precise, and reproducible technique for assessing lead exposure in meconium, which can provide a reliable paradigm for future research.

### Limitations

No previous studies have provided specific details on the extraction method of lead from meconium, not least using the ICP-MS analytic process. The present article describes the extraction method in detail, with explicit information on recovery rates using spiked and non-spiked samples. However, the main challenge with the estimation of lead in meconium was ensuring that this bio-matrix was collected reliably, especially, when it is one of a sequence of matched samples. The process of identifying recruited participants prior to giving birth, for the collection of meconium, proved to be challenging in view of the demanding clinical caseload of midwives working on a busy delivery suite. However, this problem has been resolved by placing a “study sticker” attached at the top of the maternity file [21]. Moreover, given its high sensitivity, this study should offer greater confidence to other researchers in their findings, provided that they adhere strictly to the methodology described herein.

## Ethics statements

Ethical approval through the Integrated Research Application System (IRAS) from the Leeds (East) Yorkshire and Humber Research Ethics Committee was granted (REC reference: 10/H1306/32) for this study. Detail recruitment process of the participants with signed informed consent and collection process of meconium from the participants had been described by Neelotpol *et al.,* (2016) [17]. The laboratory tests had been conducted at the LTHT NHS, Trace Elements Section, Morley, Leeds, UK.

## CRediT author statement

Conceptualization and study design: AH, MW; Sample collection: SN; Laboratory analysis: SN; Writing-Original draft: SN, MW; Fig. generation: SN, MW; Writing-review and editing: SN, AH, MW; All authors revised the manuscript, approved the final submitted version, agreed to be personally accountable for their contributions, and will ensure that any concerns regarding integrity or accuracy are investigated and resolved.

## Declaration of interests

The authors declare that they have no known competing financial interests or personal relationships that could have appeared to influence the work reported in this paper.

## Data Availability

Data will be made available on request.
